# The first invasive *Candida auris* infection in Taiwan

**DOI:** 10.1080/22221751.2022.2100280

**Published:** 2022-07-27

**Authors:** Yu-Te Tsai, Po-Liang Lu, Hung-Jen Tang, Chung-Hao Huang, Wei-Chun Hung, Yi-Ting Tseng, Kun-Mu Lee, Shang-Yi Lin

**Affiliations:** a Division of Infectious Diseases, Department of Internal Medicine, Kaohsiung Medical University Hospital, Kaohsiung, Taiwan; b Graduate Institute of Medicine, College of Medicine, Kaohsiung Medical University, Kaohsiung, Taiwan; c College of Medicine, Kaohsiung Medical University, Kaohsiung, Taiwan; d Department of Medicine, Chi Mei Medical Center, Tainan, Taiwan; e Department of Health and Nutrition, Chia Nan University of Pharmacy and Science, Tainan, Taiwan; f Department of Microbiology and Immunology, College of Medicine, Kaohsiung Medical University, Kaohsiung, Taiwan; g Department of Medical Research, Kaohsiung Medical University Hospital, Kaohsiung, Taiwan; h Department of Laboratory Medicine, Kaohsiung Medical University Hospital, Kaohsiung, Taiwan

**Keywords:** *Candida auris*, candidemia, epidemiology, South Asian clade, antifungal susceptibility, Taiwan

## Abstract

*Candida auris*, a multidrug resistant pathogenic yeast, has spread worldwide and caused several outbreaks in healthcare settings. Here, we report the first case of *C. auris* candidemia in Taiwan in a patient with a two-month history of hospitalization in Vietnam. We performed further investigation on the isolate from the present case as well as the previously reported *C. auris* isolate identified from a wound in 2018 in Taiwan, which was the first case reported in Taiwan. Both *C. auris* isolates were found to be susceptible to fluconazole, amphotericin B, and echinocandins. Additionally, mutations in *ERG11* or *FKS1* were not detected in either isolate. Microsatellite genotyping revealed that both isolates belonged to the South Asian clade. In recent years, *C. auris* has emerged as a global concern, and differences in clades and susceptibility patterns mandate further awareness and systematic surveillance.

## Introduction

*Candida auris* is an emerging pathogenic yeast that is unique because of its multidrug resistance and easily transmittable potency in nosocomial environments. *C. auris* was first identified from external ear discharge of a Japanese patient in 2009 [1], and has been documented in over 40 countries across six continents [2]. *C. auris* has resulted in several outbreaks in hospitals and other healthcare facilities [3]. It can cause invasive infections and can colonize skin and mucosa. Bloodstream infections are the most common invasive infections and are associated with high mortality rates, ranging from 30 to 60% [4,5]. Most isolates of *C. auris* are multidrug-resistant and in particular exhibit high resistance to fluconazole. Although echinocandins are the recommended regimen for treating *C. auris* infections [6], isolates resistant to all three classes of antifungal agents (azoles, polyenes, and echinocandins) have been reported [7].

*C. auris* isolates can be divided into four major clades: South Asia (I), East Asia (II), South Africa (III), and South America (IV) [8]. A potential fifth clade was reported in Iran in 2019 [9]. The genomic epidemiology of *C. auris* has been described on a global scale but little is known about its genomic epidemiology in Southeast Asia [10–12]. *C. auris* was not identified in a multicenter survey of 5064 clinical isolates of *Candida* species collected in Taiwan between 1999 and 2016, which were analyzed by DNA sequencing [13]. The first reported *C. auris* isolate in Taiwan was isolated from a patient with a superficial wound infection in 2018; however, no clade information was available [14]. Here, we report the first case of invasive *C. auris* causing candidemia in a patient with a two month history of hospitalization in Vietnam. Furthermore, we investigated the epidemiological relatedness and microbiological characteristics of the two *C. auris* isolates reported in Taiwan, the previously reported isolate [14], and the isolate from the present case.

## Materials and methods

### Laboratory investigations

Two *C. auris* isolates, one from Kaohsiung Medical University Hospital (KMUH; Kaohsiung, Taiwan) and the other from Chi Mei Medical Center (CMMC; Tainan, Taiwan), which was previously reported [14], were enrolled for analysis in the present study. The two isolates were identified by matrix-assisted laser desorption/ionization time-of-flight (MALDI-TOF MS) (Bruker Daltonik GmbH, Bremen, Germany) initially. Species identification was further confirmed by PCR following by sequencing using ITS1/ITS4 primers for the variable internal transcribed spacers (ITS1 and ITS2 regions) and 5.8SrDNA gene; and NL1/NL4 primers used to detect the D1/D2 region of the 28S ribosomal DNA(rDNA) [15,16], followed by GenBank basic local alignment search tool (BLAST) pairwise sequence alignment (http://www.ncbi.nlm.nih.gov/BLAST/Blast.cgi). The ITS and D1/D2 region sequences of two clinical isolates from the present study and *C. auris* isolates from different countries, retrieved from GenBank were included in the phylogenetic tree along with closely related *Candida* species, such as *C. haemulonii* complex and *C. pseudohaemulonii.* Antifungal susceptibility testing was performed using the broth microdilution method following the guidelines of the Clinical and Laboratory Standards Institute (CLSI) M27-S4 [17] and using the Sensititre YeastOne panel (Trek Diagnostic System, Cleveland, OH, USA). Because there are currently no established susceptibility breakpoints for *C. auris*, the minimum inhibitory concentrations (MICs) of the isolates were interpreted according to the MIC breakpoints suggested by the US Centers for Disease Control and Prevention (CDC) [18]. *ERG11* and *FKS1* gene amplification and sequencing were performed for the two isolates [16]. The sequences were analyzed using Mutation Surveyor with GenBank. The *ERG11* and *FKS1* sequences of representative strains of each genotype in this study were deposited in GenBank (Table A1). Primers for amplification the ITS, D1/D2 region, *ERG11* and *FKS1* were listed in Appendix (Table A2).

Microsatellite genotyping of *C. auris* isolates was performed using a recently developed short tandem repeat (STR) method [19]. The copy numbers of the 12 markers were determined using GeneMapper software (Applied Biosystems, Waltham, MA, USA). Relatedness between isolates was analyzed using BioNumerics software version 7.6.3 (bioMérieux, Marcy-l'Étoile, France) via the unweighted pair group method with arithmetic averages (UPGMA) using the multistate categorical similarity coefficient.

### Case presentation

A 64-year-old male patient with diabetes mellitus and prior history of ischaemic stroke was hospitalized at the University Medical Center in Ho Chi Minh City, Vietnam, in May 2021. The patient is a Taiwanese businessman who has lived in Vietnam for the past five years. Initially, he presented with disturbance of consciousness, and septic encephalopathy was suspected. While hospitalized, the patient went into cardiac arrest because of ventricular fibrillation, and he gained consciousness and spontaneous circulation was restored after resuscitation. Thereafter, he underwent renal replacement therapy because of acute kidney injury after cardiac arrest and was placed on mechanical ventilation in an intensive care unit (ICU). He received several antibacterial agents for nosocomial infections. Additionally, caspofungin was prescribed for yeast (no final identification) isolated from culture of bronchoalveolar lavage and penile ulcer pus. He was then transferred to a tertiary medical centre in Kaohsiung, Taiwan, on 10 July 2021. He had an indwelling central venous catheter (CVC) and a haemodialysis catheter. The CVC was removed on the 12th day after transfer. On the 21st day after transfer, the patient developed a fever and was hypotensive; broad-spectrum antibiotics and vasopressors (norepinephrine) were initiated. A preliminary report of blood culture yielded a yeast-like organism that was further identified as *C. auris*. The patient was started on anidulafungin (200 mg on day 1 and then 100 mg once daily) on the 26th day after transfer. Repeated blood cultures were negative on the 27th day after transfer. The treatment course for anidulafungin was 15 days. *C. auris* was not isolated from the swap samples of the skin, axilla, or groin of the patient. The patient was transferred to the respiratory care unit for weaning from ventilator. Surveillance cultures of patients in the ICU and environmental swabs were negative for *C. auris;* no additional infected or colonized cases of *C. auris* were identified at the centre.

## Results

**Figure 1.**
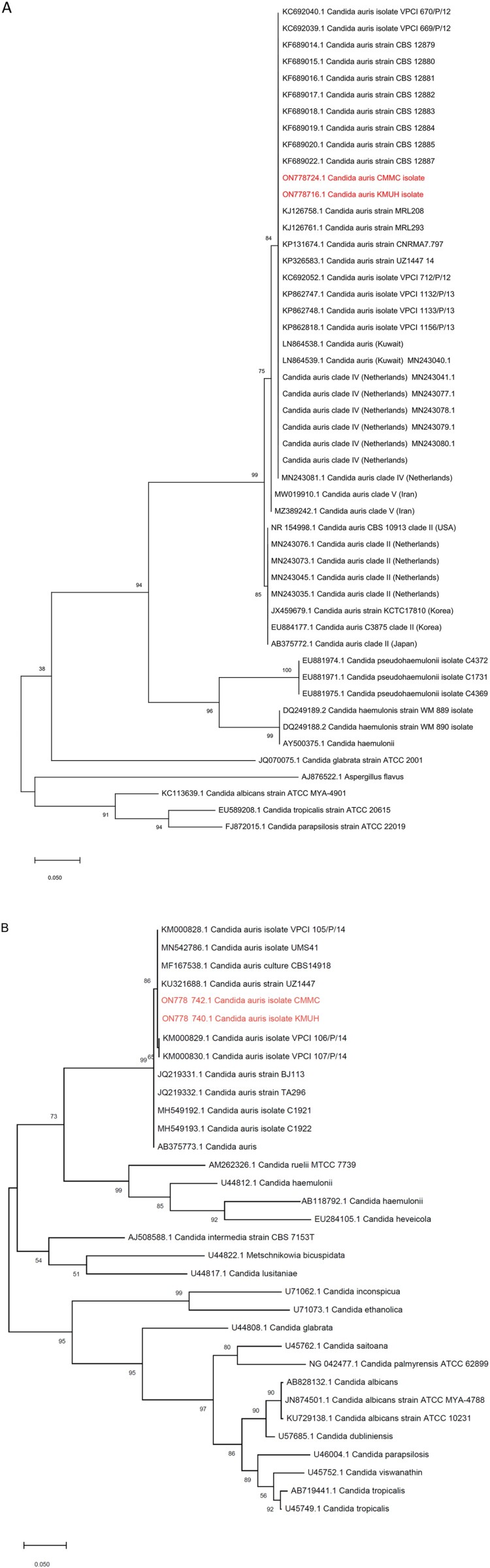
Phylogenetic tree generated by Maximum likelihood analysis using (A) ITS and (B) D1/D2 region of the *Candida auris* strains with closely related *Candida* species. The percentages of replicate trees in which the associated taxa clustered together in the bootstrap test (10,000 replicates) are indicated at the branches. The scale bar indicates the nucleotide substitutions per site. Two isolates in the present study are highlighted in red and two isolates are related to *C. auris* as it falls in the same clade.

The two isolates, KMUH and CMMC, were identified as *C. auris* by MALDI-TOF MS with log (score) values of 1.84 and 1.82, respectively. Furthermore, conclusive identification was confirmed by nucleotide sequencing of the ITS and D1/D2 regions. The ITS and D1/D2 regions phylogenetic analyses showed that the two isolates in the present study were to be identical to many other clinical strains of *C. auris* from all over the world (Figure 1(A,B)).

**Figure 2.**
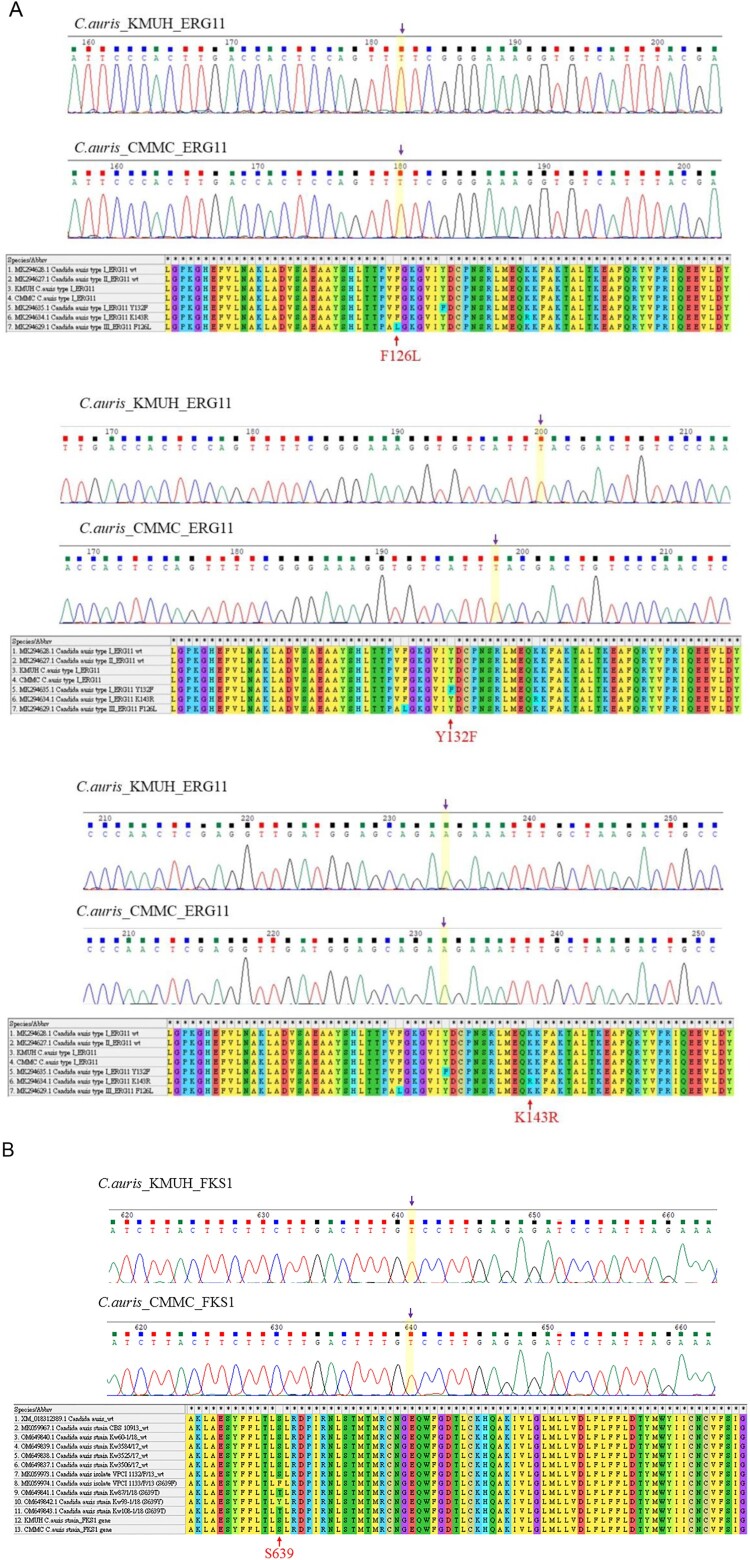
(A) Sequence alignments of *ERG11* gene in *C. auris* isolates. (B) Sequence alignments of *FKS1* hot spot 1 region in *C. auris* isolates. Mutations in *ERG11* or *FKS1* were not detected in either isolate.

**Figure 3.**
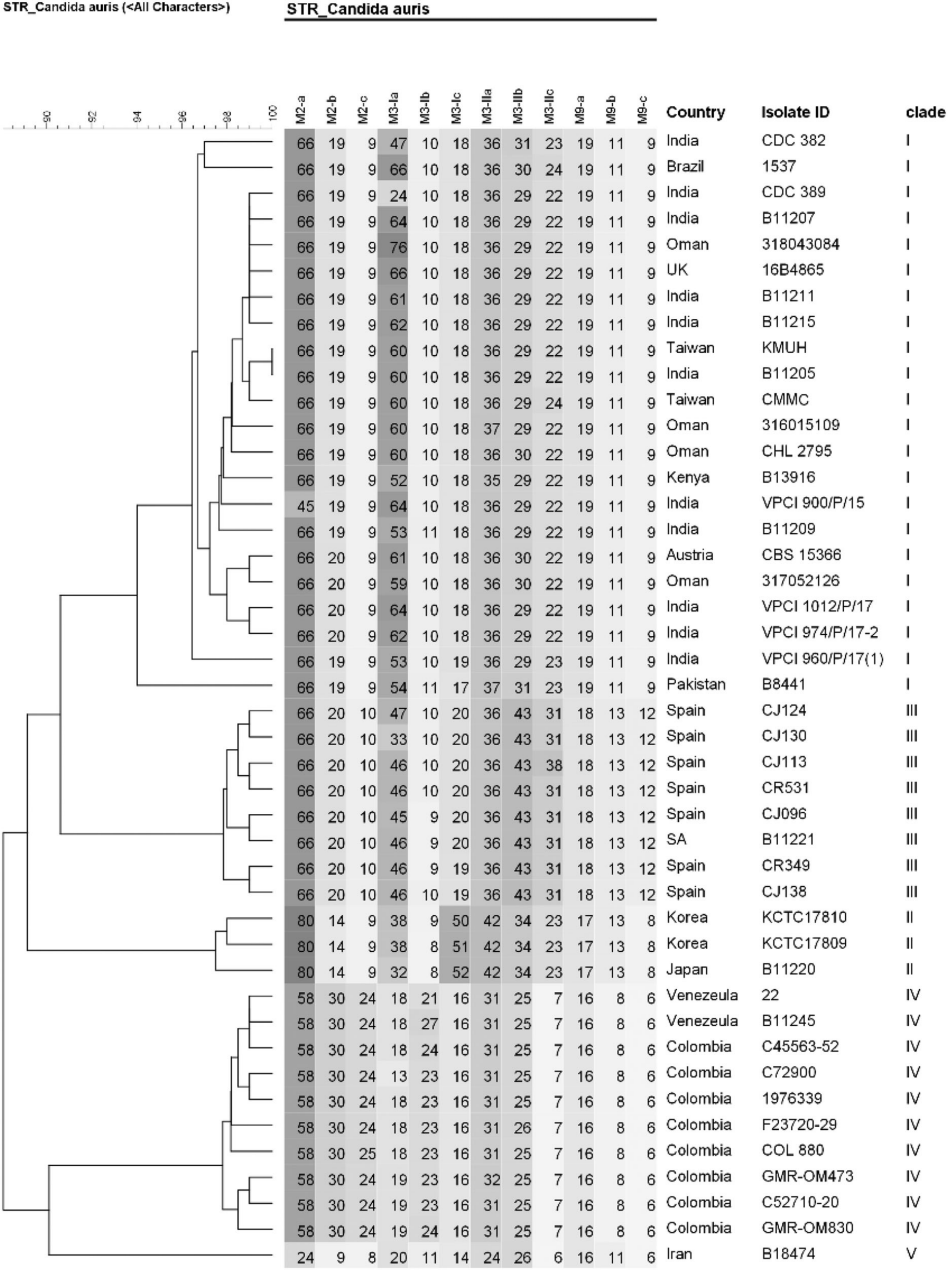
Short tandem repeat genotypes of 44 *Candida auris* isolates. Unweighted pair group method with arithmetic averages dendrogram of both isolates (KMUH and CMMC) and representative isolates from the South Asian clade and four clades are shown. Abbreviations: UK, United Kingdom; SA, South Africa.

Results of antifungal susceptibility testing are shown in Table 1. The two isolates, KMUH and CMMC, exhibited high MICs for fluconazole of 8 and 16 mg/L, respectively, and both MICs were interpreted as susceptible according to the tentative breakpoints suggested by CDC [18]. Both isolates had low MICs for amphotericin B, echinocandins (anidulafungin and micafungin), and 5-flucytosine. The two isolates were screened for F126, Y132, K143, and S639, which confer resistance to azoles and echinocandins, respectively [5]. Mutations in *ERG11* or *FKS1* were not detected in either isolate (Figure 2(A,B)). STR genotypes and UPGMA dendrogram revealed that the two isolates were closely related to the South Asian clade (I) (Figure 3). Despite being related to the South Asian clade, the CMMC isolate showed small variations (copy number 2) in the STR marker M3-IIc.

**Table 1.** Characteristics and antifungal susceptibility profiles of Candida auris isolates in Taiwan.

Isolate No.	Year of isolation	Specimen site	Antifungal MIC in mg/L (Interpretation^a^)								*ERG11* mutation^b^	*FKS1* mutation^c^
			AMB	FLC	ISA	POS	VRC	AFG	MFG	5FC		
KMUH	2021	blood	1 (S)	8 (S)	0.06 (NA)	0.12 (NA)	0.12 (NA)	0.12* (S)	0.06* (S)	≤0.06* (NA)	ND	ND
CMMC	2018	wound	0.5 (S)	16 (S)	0.25 (NA)	0.25 (NA)	0.25 (NA)	0.5-1‡ (S)	0.25-0.5‡ (S)	≤0.06 ‡ (NA)	ND	ND

Abbreviations: S, susceptible; R, resistant; NA, not applicable; AMB, amphotericin B; FLC, fluconazole; ISA, isavuconazole; POS, posaconazole; VRC, voriconazole; AFG, anidulafungin; MFG, micafungin; 5FC, 5-flucytosine; ND, not detected.

^a^ Interpretation is based on the US CDC tentative MIC breakpoints for *C. auris*: Fluconazole ≥ 32 μg/mL (R), amphotericin B ≥ 2 μg/mL (R), anidulafungin ≥ 4 μg/mL (R), micafungin ≥ 4 μg/mL (R).

^b^ Only Y132, K143, F126 were screened for azole resistance.

^c^ Only S639 was screened for echinocandin resistance.

*Performed by Sensititre YeastOne.

‡Adapted from reference of Tang et al. [14].

The sequence data for the ITS and D1/D2 regions, the ERG11 and FKS1 genes sequences were submitted to GenBank (ITS accession number: ON778716-ON778717 for KMUH isolate and ON778724-ON778725 for CMMC isolate; D1/D2 region accession number: ON778740-ON778741 for KMUH isolate and ON778742-ON778743 for CMMC isolate; ERG11 accession number: ON853792, ON853793 for KMUH and CMMC isolate, respectively; FKS1 accession number: ON853794, ON853795 for KMUH and CMMC isolate, respectively).

## Discussion

Here, we present the first case of an invasive *C. auris* infection in Taiwan. The patient had multiple risk factors [20] for susceptibility to *C. auris* infection including mechanical ventilation, prolonged ICU stay, central line indwelling, and prior antifungal exposure. The epidemiological details of the two *C. auris* cases in Taiwan were different; in our case, the patient had a recent history of prolonged hospitalization in Vietnam, whereas in the other seemed to be indigenous and the patient had no exposure to any foreign healthcare worker [14].

**Figure 4.**
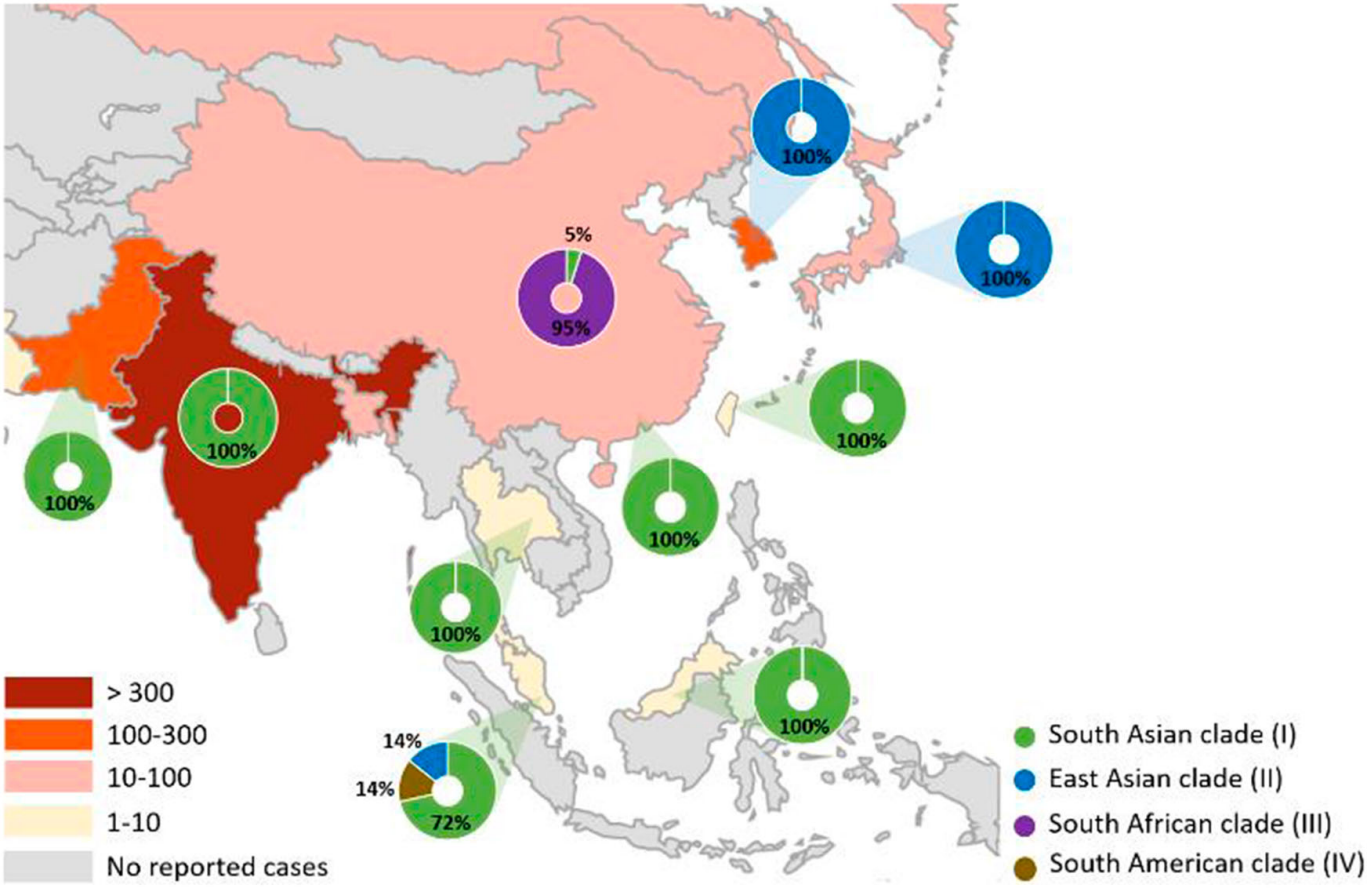
Distribution of reported *C. auris* cases in Southeast Asia and neighbouring regions/countries. Case counts were based on an epidemiological report [22] and studies from Japan [28], South Korea [29], Taiwan [14] (including the present study), China [25], Hong Kong [30], Malaysia [10], Singapore [12], Thailand [11], Bangladesh [31], and India and Pakistan [5].

Four distinct clades of *C. auris* are associated with specific geographic distribution [21–23]. In Asia, isolates from India and Pakistan mainly belong to the South Asian clade, whereas isolates from Japan and South Korea belong to the East Asian clade. Isolates from different regions of China have been reported to belong to the South Asian and South African clades [24,25]. Although several outbreaks of *C. auris* have been documented in South Asia [5,20], epidemiological reports for Southeast Asia are limited; cases have been only reported in Malaysia [10,26], Thailand [11], and Singapore [12,27]. The geographical locations of cases reported in Southeast Asia and neighbouring countries are shown in Figure 4. Tan et al. [12] reported seven cases in Singapore, and most isolates belonged to the South Asian clade. Borman et al. [32] analyzed *C. auris* isolates in the United Kingdom and compared them to strains from diverse geographic origins; the isolate reported from Malaysia (KU321688) belonged to the same lineage as India and Kuwait, which is consistent with the South Asian clade. Fifteen cases of *C. auris* colonization were reported in Hong Kong [30], and all the isolates belonged to the South Asian clade. In the present study, using STR typing, it was confirmed that the two isolates from Taiwan belonged to the South Asian clade (Figure 3). The performance of the STR-based genotyping technique is comparable to that of whole genome sequencing [19]. STR genotyping is a rapid, reliable, and cost-effective assay, and has accurate discrimination power regarding the relatedness of isolates. Several studies have used this method for epidemiological and outbreak surveys [33,34]. The KMUH isolate had the STR genotype 17, while the CMMC isolate had small variations (copy number < 5) in the STR marker M3-IIc (Figure 3). Given that persistent skin colonization of *C. auris* lasting 1–3 months, as well as environmental contamination lasting 2–3 months, have been described previously [35], we cannot exclude the possibility that our patient was already colonized with *C. auris* in Vietnam, even though only one possible *C. auris* case has been reported from Vietnam [36]. Further genomic epidemiology of *C. auris* is required in Taiwan and Vietnam.

Conventional phenotypic methods commonly misidentify *C. auris* as other Candida species, such as *Candida haemulonii, Candida famata*, *Candida catenulata*, *Candida sake*, *Rhodotorula glutinis*, and *Saccharomyces cerevisiae* [13]. Accurate identification of *C. auris* can be achieved using MALDI-TOF and molecular identification based on sequencing of ITS regions of rDNA and the D1/D2 domain of 28S rDNA [37].

MALDI-TOF MS is an efficient and reliable diagnostic tool for *C. auris*, given that it presents with reference spectra in database or research use only library (RUO) [13]. Additionally, MALDI-TOF has advantage of reduced turnaround time compared to conventional or molecular methods [38]. rDNA sequencing of ITS and 28S D1/D2 region will accurately identify *C. auris* to the species level, but it is not routinely available in clinical laboratories due to higher technical and instrumental requirements [38]. Our two isolates (KMUH and CMMC) were confirmed by MALDI-TOF MS and rDNA sequencing methods. Laboratories in Asia mainly rely on conventional phenotypic and morphological methods for identification of fungi, and very few laboratories rely on DNA sequencing (16.9%) or MALDI-TOF MS (12.3%) for isolate identification [39]. Therefore, the true burden of *C. auris* infections in Asia remains largely unknown.

Most *C. auris* isolates are resistant to fluconazole [5]. Three hot spot mutations (Y132F, K143R, and F126L) have been reported in *ERG11* in fluconazole-resistant *C. auris* strains belonging to different genetic clades [5]; however, these mutations were not detected in the present study in either isolate (Figure 2). Most South Asian clade I isolates are resistant to fluconazole and demonstrate high MICs for polyenes [40–42]. However, fluconazole-susceptible *C. auris* isolates belonging to the South Asian clade (I) with low MICs for azoles have been reported [33,43]. The two *C. auris* isolates in Taiwan had elevated MICs for fluconazole but were still susceptible to fluconazole. Majority of the South Asian clade isolates from India and Pakistan were resistant to fluconazole (97–100%) with varying resistance to polyenes (7.9–93.7%) [44,45], respectively; however, only one South Asian clade isolate in China [25,43] and two isolates in Taiwan ([14] and the present report) were susceptible to all three classes of antifungal agents. Whether a subclade exhibiting low MICs for fluconazole within the South Asian clade has circulated in Taiwan and Vietnam requires further investigation.

Travel restrictions due to the ongoing coronavirus disease (COVID-19) pandemic were expected to reduce the transmission of *C. auris* between countries. However, superinfection cases and outbreaks of *C. auris* in COVID-19 care facilities have been reported in several countries [46–49], and were associated with high mortality rates, up to 60% [46]. Owing to long-term survival of *C. auris* on inanimate surfaces, contact precautions are crucial for the prevention of *C. auris* transmission in healthcare settings [8,13]. Fortunately, in the present case, the patient was cared for in a single room with contact precautions since being transferred to our hospital according to the COVID-19 quarantine policy, and there was no indication of hospital transmission. In addition to COVID-19 screening, active surveillance for *C. auris* should be considered for high-risk patients [3,13].

## Conclusions

In summary, we presented the first case of *C. auris* candidemia in Taiwan. STR genotyping revealed that two isolates from Taiwan, the previously reported isolate and the isolate from the present case, belonged to the South Asian clade. The prevalence of *C. auris* in Southeast Asia may be underestimated, and development of quality mycology laboratories and further surveillance are required.

## Appendix

**Table A1.** GenBank accession numbers, *ERG11* and *FKS1* genotypes, and clade information of representative strains included in this study.

Strain/isolate	Clade	*ERG11*	
		Accession no.	Genotype
AR0382	South Asian	MK294628	WT
AR0381	East Asian	MK294627	WT
AR0389	South Asian	MK294635	Y132F
AR0388	South Asian	MK294634	K143R
AR0383	South African	MK294629	F126L
Strain/isolate	Clade	*FKS1*	
		Accession no.	Genotype
Reference		XM_018312389	WT
CBS 10913	East Asian	MK059967	WT
KW60/18		OM649840	WT
KW3584/17		OM649839	WT
KW3525/17		OM649838	WT
KW3506/17		OM649837	WT
VPCI 1132/*P*/13	South Asian	MK059973	WT
VPCI 1133/*P*/13	South Asian	MK059974	S639F
KW87/1/18		OM649841	S639T
KW93/18		OM649842	S639Y
KW108/18		OM649843	S639T

WT, wild type.

**Table A2.** Sequences of primers used in the study.

Gene	Primer probe		Sequence
ITS	ITS1	Forward	5’-TCC GTA GGT GAA CCT GCG G-3’
	ITS4	Reverse	5’-TCC TCC GCT TAT TGA TAT GC-3’
D1/D2 region	NL-1	Forward	5’-GCA TAT CAA TAA GCG GAG GAA AAG-3’
	NL-4	Reverse	5’-GGT CCG TGT TTC AAG ACG G-3’
ERG11 (Amplify)	CauErg11F	Forward	5’-GTG CCC ATC GTC TAC AAC CT-3’
	CauErg11R	Reverse	5’-TCT CCC ACT CGA TTT CTG CT-3’
ERG11 (Sequencing)	CauERG11dF	Forward	5’-TGG GTK GGY TCW GCT GTT G-3’
	CauERG11dR	Reverse	5’-TTC WGC TGG YTC CAT TGG-3’
FKS1 (Amplify)	CauFKS1_F	Forward	5’-ATG TCT TAC GAT AAC AAT C-3’
	CauFKS1_R	Reverse	5’-TTA GAA TGC CTT TGT AGT ATA G-3’
FKS1 (Sequencing)	CauFKS1_F1256-77	Forward	5’-AGA GAT ACA TGA GAT TGG GTG-3’
		Reverse	

ITS: internal transcribed spacers.
